# End-of-Life Care Among Patients With Kidney Failure on Maintenance Dialysis: A Retrospective Population-Based Study

**DOI:** 10.1177/20543581241280698

**Published:** 2024-09-21

**Authors:** Shuaib Hafid, Sarina R. Isenberg, Aleisha Fernandes, Erin Gallagher, Colleen Webber, Meera Joseph, Manish M Sood, Adrianna Bruni, Janet L. Davis, Grace Warmels, James Downar, Anastasia Gayowsky, Aaron Jones, Doug Manuel, Peter Tanuseputro, Michelle Howard

**Affiliations:** 1 Department of Family Medicine, McMaster University, Hamilton, ON, Canada; 2 Department of Medicine, University of Ottawa, ON, Canada; 3 Bruyère Research Institute, University of Ottawa, ON, Canada; 4 Ottawa Hospital Research Institute, The Ottawa Hospital, University of Ottawa, ON, Canada; 5 Division of Nephrology, Department of Medicine, McMaster University, Hamilton, ON, Canada; 6 Department of Medicine, Ottawa Hospital Research Institute, The Ottawa Hospital, University of Ottawa, ON, Canada; 7 Division of Palliative Care, Department of Medicine, Ottawa Hospital Research Institute, The Ottawa Hospital, University of Ottawa, ON, Canada; 8 ICES McMaster, McMaster University, Hamilton, ON, Canada; 9 Department of Health Research Methods, Evidence, and Impact, McMaster University, Hamilton, ON, Canada; 10 Department of Family Medicine, University of Ottawa, ON, Canada; 11 ICES uOttawa, University of Ottawa, ON, Canada

**Keywords:** cohort studies, dialysis, outpatient care, primary care, palliative care

## Abstract

**Background:**

Nephrologists routinely provide end-of-life care for patients with kidney failure (KF) on maintenance dialysis. Involvement of primary care and palliative care physicians may enhance this experience.

**Objective:**

The objective was to describe outpatient care patterns in the last year of life and the end-of-life acute care utilization for patients with KF on maintenance dialysis.

**Design:**

Retrospective cohort study using population-level health administrative data.

**Setting & Participants:**

Outpatient and inpatient care during the last year of life among patients who died between 2017 and 2019, receiving maintenance dialysis in Ontario, Canada.

**Measurements:**

The primary exposure is patterns of physician specialties providing outpatient care in the last year of life. Outcomes include outpatient encounters in the last year of life, acute care visitation in the last month of life, and place of death.

**Methods:**

We reported the count and percentage of categorical outcomes and the median (interquartile range) for numeric outcomes. We produced time series plots of the mean monthly percentage of encounters to different specialties stratified by physician specialty patterns. We evaluated differences in outcomes by physician specialty patterns using analysis of variance (ANOVA) and Pearson’s chi-square tests (*P* < .05, two-tailed).

**Results:**

Among 6866 patients, the median age at death was 73, 36.1% were female, and 87.8% resided in urban regions. Three patterns emerged: a primary care, nephrology, and palliative care triad (25.5%); a primary care and nephrology dyad (59.3%); and a non-primary care pattern (15.2%). Palliative care involvement is concentrated near death. Of all, 81.4% spent at least 1 day in hospital or emergency department in the last month, but those with primary care, palliative care, and nephrology involvement had the fewest acute care deaths (65.8%).

**Limitations:**

Outpatient care patterns were defined using physician billing codes, potentially missing care from other providers.

**Conclusions:**

Nephrology and primary care predominantly manage outpatient care in the last year of life for patients with KF on maintenance dialysis, with consistent acute care use across care patterns except for the place of death. Future research should explore associations between patterns of care and end-of-life outcomes to identify the most optimal model of care for patients with KF on maintenance dialysis.

## Abrégé

**Contexte:**

Il est courant pour les néphrologues de prodiguer des soins de fin de vie aux patients souffrant d’insuffisance rénale (IR) sous dialyse d’entretien. Cette expérience pourrait être enrichie par la participation des médecins des unités de soins primaires et de soins palliatifs.

**Objectif:**

Cette étude visait à décrire les modèles de soins ambulatoires prodigués au cours de la dernière année de vie et l’utilisation des soins aigus en fin de vie chez les patients atteints d’IR sous dialyse d’entretien.

**Conception:**

Étude de cohorte populationnelle rétrospective réalisée à partir des données administratives du système de santé.

**Cadre et sujets de l’étude:**

Les soins ambulatoires et hospitaliers au cours de la dernière année de vie chez les patients décédés sous dialyse d’entretien entre 2017 et 2019 en Ontario (Canada).

**Mesures:**

La principale mesure est le profil des spécialités médicales qui fournissent des soins ambulatoires dans la dernière année de vie. Les données recueillies comprennent les consultations externes au cours de la dernière année de vie, les visites en soins aigus au cours du dernier mois de vie et le lieu du décès.

**Méthodologie:**

Nous avons rapporté le nombre et le pourcentage de résultats catégoriels, ainsi que la médiane (écart interquartile) des résultats numériques. Nous avons produit des graphiques chronologiques du pourcentage mensuel moyen de consultations avec différentes spécialités, stratifiées selon les spécialités médicales. Nous avons évalué les différences dans les résultats selon les profils de spécialités médicales en utilisant les tests ANOVA et Chi-Square de Pearson (*P* <,05; bilatéral).

**Résultats:**

Des 6 866 patients inclus (âge médian au décès: 73 ans), 36,1% étaient des femmes et 87,8% vivaient en région urbaine. Trois modèles sont apparus: une triade soins primaires, néphrologie et soins palliatifs (25,5%); une dyade soins primaires et néphrologie (59,3%); et un modèle de soins non primaires (15,2%). La participation des soins palliatifs est concentrée autour du moment du décès. Une grande majorité des patients (81,4%) avait passé au moins une journée à l’hôpital ou aux urgences au cours du dernier mois, mais les personnes qui avaient bénéficié d’une triade de soins (primaires, néphrologie et soins palliatifs) présentaient une moins grande proportion de décès en soins aigus (65,8%).

**Limites:**

Les modèles de soins ambulatoires ont été définis à l’aide des codes de facturation des médecins, ce qui pourrait avoir exclu les soins dispensés par d’autres prestataires.

**Conclusion:**

Les soins ambulatoires au cours de la dernière année de vie des patients atteints d’IR sous dialyse d’entretien sont principalement prodigués par la néphrologie et les soins primaires, avec une utilisation constante des soins aigus dans tous les modèles de soins, sauf pour le lieu du décès. Les futures recherches devraient explorer les liens entre les modèles de soins et les résultats en fin de vie afin d’identifier le modèle de soins le plus optimal pour les patients atteints d’IR sous dialyse d’entretien.

## Background

Patients with kidney failure experience high rates of mortality even if they receive dialysis. It is common for patients with kidney failure (KF) on maintenance dialysis to experience serious acute health events such as myocardial infarctions, and 3 out of 5 patients on dialysis experience at least one emergency department visit in the last month of life.^1^ Patients with KF on maintenance dialysis will receive routine life-sustaining dialysis sessions, while also experiencing high symptom burden; this combination may be debilitating for patients approaching the end of life. Most patients with KF on maintenance dialysis do not receive palliative care from palliative care physicians despite experiencing a terminal illness,^1^ and care from palliative care physicians is typically concentrated within the last 30 days of life for those who receive it.^2^ This trend aligns with other studies of patients dying of organ failure, who receive fewer palliative care services compared to patients dying of cancer.^3^

Patients with KF on maintenance dialysis receive regular care and high continuity with their nephrology team.^4^ In addition, they experience a significant burden from co-morbid conditions,^5,6^ thus patients may have multiple physician specialties involved in their care and a regular primary care provider. Research from Ontario, Canada, found that high continuity with a primary care physician before and during dialysis initiation was not associated with decreased mortality risk or hospitalizations,^7^ while a study from Alberta, Canada, identified that poor continuity of care with primary care was associated with increased acute care utilization patterns among patients with stage 3 or 4 chronic kidney disease.^8^ Neither study focused on utilization patterns during the last year of life, and therefore, their findings may not reflect the end-of-life context regardless of the conflicting results.

Involving multiple specialties to care for different conditions may unintentionally undermine the known benefits of continuity of care. Primary care’s role among patients with KF on maintenance dialysis during the last year of life has yet to be extensively studied, despite, primary care being responsible for most of the outpatient care in the last year of life among the general population.^9^ In addition, there is also evidence that an increase in continuity with primary care during end of life is associated with decreased acute care utilization.^10-14^ However, exactly which specialties of physicians they see, and how often they see them is unknown for patients dying with KF on maintenance dialysis. Further, the mix, timing, and intensity of physician care in the community and its relation to end-of-life healthcare utilization outcomes have not been previously described for this population. Hence, we examined the physician care team and the location of death among individuals with KF on maintenance dialysis during the last year of life.

## Methods

### Study Design and Data Sources

We conducted a retrospective cohort study using linked population-level health administrative data in Ontario, Canada (see Supplementary File 1 for a description of the datasets accessed). Administrative datasets are linked using unique encoded identifiers and analyzed at ICES (formerly known as the Institute for Clinical Evaluative Sciences).

### Study Cohort

Our cohort included adult patients who died between January 1, 2017, and December 31, 2019, and who had any record of long-term maintenance dialysis initiation (including peritoneal dialysis) before their death date in the Canadian Organ Replacement Register (CORR) (see Supplementary File 1 for a description of data sources accessed). We excluded patients who were aged >105 years at death (in case of documentation errors in the patients’ birth or death dates) and those who were ineligible for the Ontario Health Insurance Plan (OHIP) at any point in the last year of life. Other exclusion criteria included patients who resided in a long-term care home in the last year of life, as these individuals receive different care patterns to patients residing in the community, and patients who did not have any weekly dialysis billing codes in the last year of life.

### Physician Care Patterns

Our study exposure examines the mix of care provided by different physician specialties in the last year of life. Specifically, we focused on nephrology due to the nature of the population of interest, palliative care due to the end-of-life context, and primary care as primary care is responsible for most outpatient care provision in the last year of life for the general population.^9^ We defined mutually exclusive categories of different outpatient physician patterns of care during the last year of life (Figure 2). First, patients who did not have an outpatient encounter with a primary care physician in the last year of life were categorized into the non-primary care pattern of care. Patients in this category may have any combination of encounters with all other specialties. Patients who did have an outpatient encounter with a primary care physician were categorized into the following categories: (1) primary care, nephrology, and palliative care triad and (2) primary care and nephrology care dyad. Patients were categorized into the care triad if they had at least one encounter with a primary care physician, nephrologist, and palliative care physician in the last year of life. Similarly, patients were categorized into the care dyad if they had at least one encounter with primary care and nephrology but did not have any palliative care encounters.

Physician specialty was obtained through OHIP billing records for all specialties except for palliative care as any physician can bill palliative care codes regardless of their specialty. We adapted a previously defined algorithm that is highly sensitive and has positive predictive value in identifying self-reported palliative care physicians.^15^ The algorithm identified palliative care physicians as physicians with greater than 10% of their billings over 2 years as palliative care. We applied this definition to physicians who billed for palliative care services during the first calendar year of our study period (January 1, 2017, to December 31, 2017) and looked back 2-years before each of the unique palliative care billing codes to identify an average percentage of their billing patterns.

### Outcomes

The outcomes of interest capture outpatient and inpatient care patterns in the last year of life. First, we measured the number of outpatient physician encounters by the outpatient physician care pattern categories in the last year of life, using individual outpatient billing codes in OHIP. We identified that this approach was inappropriate for nephrologist encounters who typically bill weekly maintenance dialysis billing codes and do not bill for each encounter. Hemodialysis patients will typically receive dialysis 3 times per week, wherein they interact with their dialysis care team, which can include the nephrologist and registered staff. As such, we supplemented the original definition by approximating outpatient encounters with nephrologists by the number of weekly maintenance dialysis OHIP billing codes (Supplementary File 2).

Next, we measured how end-of-life acute care utilization varies by patterns of physician care. Specifically, we examined the number of unique hospitalizations or emergency department visits in the last 30 days of life, the number of days spent in hospital or emergency department in the last 30 days of life, and the percentage of patients that died in acute care settings. We also reported patients’ acute care utilization in the last year of life, truncating the last 30 days of life. We used the Discharge Abstract Database and the National Ambulatory Care Reporting System to capture inpatient hospitalization and the Registered Persons Database to capture patient death locations: acute (i.e., emergency departments and inpatient hospital settings), sub-acute (i.e., complex continuing care and rehabilitation) or other (i.e., primarily in the community).^16^

### Patient Characteristics

Patient sociodemographic and clinical characteristics were reported. Neighborhood-level income and rurality status were identified using the patients’ postal code one year before their death date. Prevalence of chronic conditions was obtained using a 5-year look back period from the patients’ death date, using previously developed algorithms to assign the prevalence of 18 conditions.^17-25^ Comorbidity status at 1 year before death was also captured using the Johns Hopkins Adjusted Clinical Group (ACG)® System Aggregated Diagnosis Groups, Version 10.^26^ All ICD-9/ICD-9-CM diagnosis codes assigned to patients are categorized into 32 functional groups, based on the diagnostic certainty, duration, severity, and etiology of the condition, and the likelihood of requiring specialist care.^27^ Dialysis location, type, and duration of time between dialysis initiation and death were captured using the CORR. Patients’ primary care physician roster status was captured using the Client Agency Program Enrolment table for patients formally attached to a primary care physician and the rostered primary care physician was the primary care physician with the highest proportion of encounters in the last year of life, for patients not formally attached.^28^

### Analyses

Descriptive results were presented as proportions for categorical variables and as mean and standard deviation (SD), or medians and interquartile ranges (IQR) for skewed continuous variables. We reported study outcomes, stratified by the exposure variable, by producing means, SDs, medians, and IQRs. We also produced a time series plot reporting monthly percentages of outpatient encounters by physician specialties in the last year of life. We measured health utilization differences across patterns by conducting an Analysis of Variance (ANOVA) for numeric variables and Pearson’s chi-square tests for categorical variables. Statistical significance was set at *P*-value <.05 (two-tailed). All analyses were completed using SAS Enterprise Guide v. 7.15.

### Ethics Approval

This study was approved by the Hamilton Integrated Research Ethics Board on March 28, 2022 (#14750-C). ICES is an independent, non-profit research institute whose legal status under Ontario’s health information privacy law (section 45 of Ontario’s Personal Health Information Protection Act) allows it to collect and analyze healthcare and demographic data, without consent, for health system evaluation and improvement.

## Results

**Figure 1.**
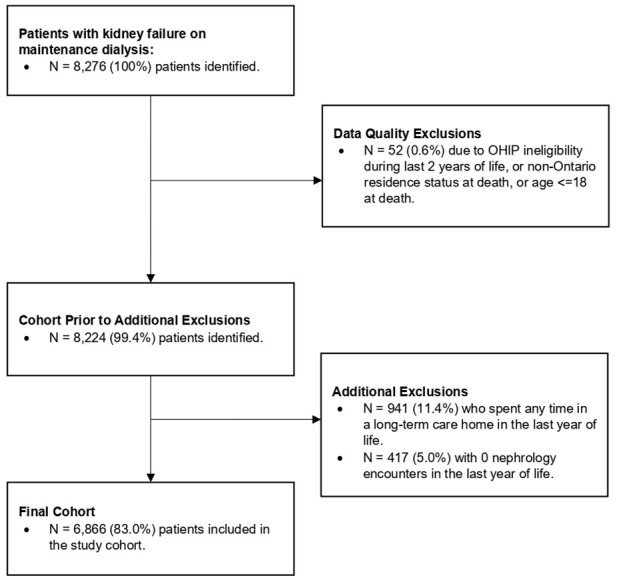
Cohort creation flowchart.

**Figure 2.**
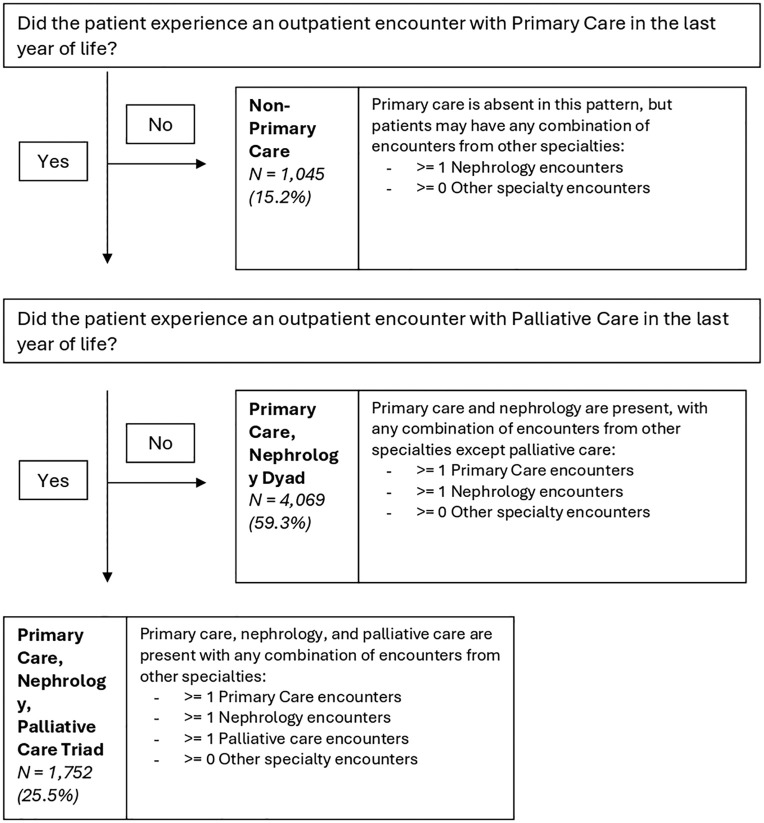
Patterns of outpatient physician care experienced by patients dying with kidney failure on maintenance dialysis between January 1, 2017, and December 31, 2019, in Ontario, Canada.

After applying the exclusion criteria, the cohort included 6,866 patients (Figure 1). Patients had a median age of 73 (64-81) at death, 36.1% were female, and 87.8% resided in urban regions (Table 1). 25.5% of patients experienced care from a primary care, nephrology, and palliative care triad; 59.3% experienced care from a primary care and nephrology dyad, while 15.2% experienced a non-primary care pattern in the last year of life (Figure 2). Dialysis modality and location did not differ across patterns of care, with approximately 70% of patients experiencing hemodialysis in-center. Patients who received care from a primary care, palliative care, and nephrology triad had a higher prevalence of cancer at 1-year before death (40.8%), compared to patients who received care from a primary care and nephrology dyad (24.1%), or patients who received the non-primary care pattern (19.2%). In addition, patients who received primary care, palliative care, and nephrology triad had the highest percentage of receiving provincial home care services in the last year of life (89.4%), compared to patients who experienced a primary care and nephrology dyad (77.7%) or a non-primary care pattern (74.7%). Patients who experienced a non-primary care pattern also had the greatest percentage of patients in the lowest neighborhood income quintile (34.1%) compared to the primary care and nephrology dyad (27.2%) and the primary care, palliative care, and nephrology care triad (26.2%).

**Table 1.** Profile of Patients Aged 19 or Older With Kidney F﻿ailure on Maintenance Dialysis Who Died Between January 1, 2017, and December 31, 2019, in Ontario, Canada, Reported by Physician Care Patterns.

Variable	Total Cohort	Pattern: Primary care, palliative care, nephrology triad	Pattern: Primary care, nephrology dyad	Pattern: Non-primary care	*P*-value
	N = 6866	N = 1752	N = 4069	N = 1045	
Age at death, N (%)					
19 to 44	204 (3.0%)	32 (1.8%)	126 (3.1%)	46 (4.4%)	<.001
45 to 54	456 (6.6%)	80 (4.6%)	267 (6.6%)	109 (10.4%)	
55 to 64	1097 (16.0%)	257 (14.7%)	636 (15.6%)	204 (19.5%)	
65 to 74	1983 (28.9%)	522 (29.8%)	1207 (29.7%)	254 (24.3%)	
75 to 84	2100 (30.6%)	553 (31.6%)	1259 (30.9%)	288 (27.6%)	
85 to 94	987 (14.4%)	292 (16.7%)	561 (13.8%)	134 (12.8%)	
95 to 104	39 (0.6%)	16 (0.9%)	13 (0.3%)	10 (1.0%)	
Median (IQR)	73 (64-81)	74 (66-82)	73 (64-81)	71 (60-80)	<.001
Female, N (%)	2480 (36.1%)	646 (36.9%)	1443 (35.5%)	391 (37.4%)	.337
Urban residence, N (%)	6028 (87.8%)	1609 (91.8%)	3485 (85.7%)	934 (89.4%)	<.001
Income quintile at 1-year before death, N (%)					
1 (lowest)	1920 (28.0%)	459 (26.2%)	1105 (27.2%)	356 (34.1%)	<.001
2	1542 (22.5%)	391 (22.3%)	910 (22.4%)	241 (23.1%)	
3	1304 (19.0%)	338 (19.3%)	797 (19.6%)	169 (16.2%)	
4	1072 (15.6%)	267 (15.2%)	658 (16.2%)	147 (14.1%)	
5 (highest)	995 (14.5%)	291 (16.6%)	586 (14.4%)	118 (11.3%)	
Missing	33 (0.5%)	6 (0.3%)	13 (0.3%)	14 (1.3%)	
Adjusted diagnostic groups at 1-year before death,					
Median (IQR)	12 (9-15)	12 (9-15)	12 (9-15)	12 (8-14)	<.001
Prevalence of cancer at 1-year before death, N (%)	1895 (27.6%)	714 (40.8%)	980 (24.1%)	201 (19.2%)	<.001
Prevalence of heart failure at 1-year before death, N (%)	2413 (35.1%)	582 (33.2%)	1502 (36.9%)	329 (31.5%)	<.001
Duration of time (in years) between dialysis initiation and death date,					
Mean (SD)	4.8 (5.9)	4.6 (5.9)	4.6 (5.8)	6.1 (6.5)	<.001
Dialysis modality, N (%)					
Only hemodialysis	4846 (70.6%)	1279 (73.0%)	2829 (69.5%)	738 (70.6%)	<.001
Only peritoneal dialysis	757 (11.0%)	194 (11.1%)	473 (11.6%)	90 (8.6%)	
Both hemodialysis and peritoneal dialysis	1263 (18.4%)	279 (15.9%)	767 (18.9%)	217 (20.8%)	
Dialysis location, N (%)					
In-center	4694 (68.4%)	1254 (71.6%)	2740 (67.3%)	700 (67.0%)	.002
Home	109 (1.6%)	23 (1.3%)	77 (1.9%)	9 (0.9%)	
Both in-center and home	2063 (30.1%)	475 (27.1%)	1252 (30.8%)	336 (32.2%)	
Received provincial home care services in last year of life, N (%)	5508 (80.2%)	1566 (89.4%)	3161 (77.7%)	781 (74.7%)	<.001
Rostered to a primary care physician, N (%)	6560 (95.5%)	1712 (97.7%)	3977 (97.7%)	871 (83.4%)	<.001

Patients had a median of 51 (IQR: 16-53) outpatient encounters with nephrologists in the last year of life, regardless of which pattern they experienced (Table 2). Patients who experienced patterns of care that involved primary care experienced a median of 5 outpatient encounters with primary care physicians in the last year of life. Patients who experienced a primary care, palliative care, and nephrology triad experienced a median of 3 outpatient encounters with palliative care in the last year of life. Only 27.4% of patients who received a non-primary care pattern had an encounter with a palliative care physician in the last year of life.

**Table 2.** Outpatient Encounter Patterns During the Last Year of Life Among Patients Dying With Kidney F﻿ailure on Maintenance Dialysis Between January 1, 2017, and December 31, 2019, in Ontario, Canada.

Outcome	Total cohort	Pattern: Primary care, palliative care, nephrology triad	Pattern: Primary care, nephrology dyad	Pattern: Non-primary care	*P*-value
	N = 6866	N = 1752	N = 4069	N = 1045	
Primary Care:					
Number of patients with ≥1 encounter, N (%)	5821 (84.8%)	1752 (100.0%)	4069 (100.0%)	0 (0.0%)	<.001
Number of outpatient encounters, Median (IQR)	4 (1-7)	5 (2-9)	5 (2-8)	0 (0-0)	<.001
Palliative Care:					
Number of patients with ≥1 encounter, N (%)	2038 (29.7%)	1752 (100.0%)	0 (0.0%)	286 (27.4%)	<.001
Number of outpatient encounters, Median (IQR)	0 (0-1)	3 (2-7)	0 (0-0)	0 (0-1)	<.001
Nephrology:					
Number of patients with ≥1 encounter, N (%)	6866 (100.0%)	1752 (100.0%)	4069 (100.0%)	1045 (100.0%)	-
Number of outpatient encounters, Median (IQR)	51 (16-53)	49 (9-52)	51 (16-53)	52 (44-53)	<.001
Oncology:					
Number of patients with ≥1 encounter, N (%)	661 (9.6%)	376 (21.5%)	212 (5.2%)	73 (7.0%)	<.001
Number of outpatient encounters, Median (IQR)	0 (0-0)	0 (0-0)	0 (0-0)	0 (0-0)	<.001
Internal Medicine:					
Number of patients with ≥1 encounter, N (%)	4759 (69.3%)	1217 (69.5%)	2839 (69.8%)	703 (67.3%)	.291
Number of outpatient encounters, Median (IQR)	2 (0-10)	2 (0-8)	2 (0-11)	2 (0-17)	.019
Other Specialties:					
Number of patients with ≥1 encounter, N (%)	6337 (92.3%)	1647 (94.0%)	3809 (93.6%)	881 (84.3%)	<.001
Number of outpatient encounters, Median (IQR)	6 (3-11)	7 (3-11)	6 (3-11)	4 (1-8)	<.001

Almost all patients (92.3%) experienced an encounter with other specialties in the last year of life, with a median of 6 (IQR: 3-11) outpatient encounters.

**Figure 3.**
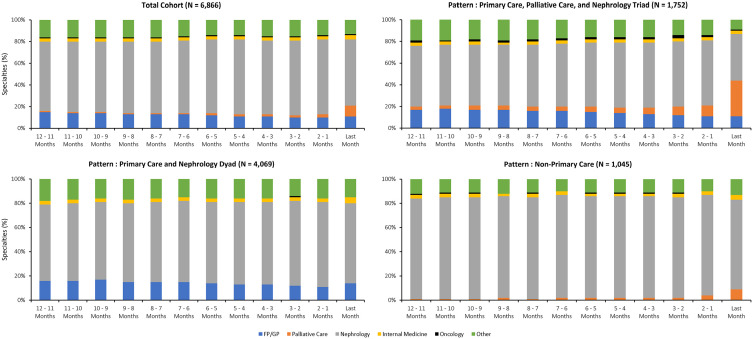
Individual-level monthly percentages of outpatient encounters to different specialties during the last year of life among patients dying with kidney failure on maintenance dialysis between January 1, 2017, and December 31, 2019, in Ontario, Canada.

Time series plots of the monthly percentages of outpatient encounters to different specialties identified primary care plays a consistent role in outpatient care provision in the last year of life, in addition to the consistent care provision by nephrologists (Figure 3, Supplementary File 3). Among patients who received care from the primary care, palliative care, and nephrology triad and those who received care from the non-primary care pattern, palliative care involvement progressively increased in the last 3 months of life. Regardless of pattern, almost 20% of monthly encounters were attributed to specialties other than primary care, palliative care, or nephrology.

Patients who received care from the primary care, palliative care, and nephrology triad had the lowest percentages of any hospitalization (55.7%) or any emergency department visits (51.8%) in the last 30 days of life (Table 3), compared to patients who received other patterns of care. Regardless of these differences, all patterns experienced a median of 1 (IQR: 0-1) hospitalization and a median of 1 (IQR: 0-1) emergency department visit in the last 30 days of life. Despite experiencing the lowest percentages of hospitalizations or emergency department visits, patients who received care from the primary care, palliative care, and nephrology triad had the highest number of days spent in the hospital or emergency department (median 13 [IQR: 3-26]) compared to patients who experienced a primary care and nephrology dyad (median 8 [IQR: 1-22]) or a non-primary care pattern (median 8 [1-23]). Patients who received care from a primary care, palliative care, and nephrology pattern had the lowest percentage of deaths in acute care settings (65.8%), compared to patients who experienced a primary care and nephrology dyad (80.2%) or a non-primary care pattern (74.5%). Differences in acute care utilization during the last month of life across patterns were statistically significant (<.05).

**Table 3.** End-of-life healthcare outcomes among patients dying with kidney failure on maintenance dialysis between January 1, 2017, and December 31, 2019, in Ontario, Canada.

Variable	Value	Total cohort	Pattern: Primary care, palliative care, nephrology triad	Pattern: Primary care, nephrology dyad	Pattern: Non-primary care	*P*-value
		N = 6866	N = 1752	N = 4069	N = 1045	
Inpatient hospitalization in the last year of life (truncating the last 30 days of life)	Any, N (%)	5252 (76.5%)	1477 (84.3%)	3055 (75.1%)	720 (68.9%)	<.001
	Median (IQR)	2 (1-3)	2 (1-3)	2 (1-3)	1 (0-3)	<.001
Inpatient hospitalization in the last 30 days of life	Any, N (%)	4092 (59.6%)	975 (55.7%)	2517 (61.9%)	600 (57.4%)	<.001
	Median (IQR)	1 (0-1)	1 (0-1)	1 (0-1)	1 (0-1)	<.001
Emergency department visit in the last year of life (truncating the last 30 days of life)	Any, N (%)	5955 (86.7%)	1605 (91.6%)	3531 (86.8%)	819 (78.4%)	<.001
	Median (IQR)	3 (1-5)	3 (2-5)	3 (1-5)	2 (1-4)	<.001
Emergency department visit in the last 30 days of life	Any, N (%)	3862 (56.3%)	908 (51.8%)	2383 (58.6%)	571 (54.6%)	<.001
	Median (IQR)	1 (0-1)	1 (0-1)	1 (0-1)	1 (0-1)	<.001
Number of days spent in hospital or emergency department in last year of life (truncating the last 30 days of life)	Any, N (%)	6120 (89.1%)	1638 (93.5%)	3623 (89.0%)	859 (82.2%)	<.001
	Median (IQR)	18 (4-48)	26 (8-56)	16 (4-44)	15 (2-49)	<.001
Number of days spent in hospital or emergency department in last 30 days of life	Any, N (%)	5591 (81.4%)	1458 (83.2%)	3304 (81.2%)	829 (79.3%)	.032
	Median (IQR)	9 (1-24)	13 (3-26)	8 (1-22)	8 (1-23)	<.001
Place of death	Acute	5195 (75.7%)	1153 (65.8%)	3263 (80.2%)	779 (74.5%)	<.001
	Sub-acute	414 (6.0%)	198 (11.3%)	135 (3.3%)	81 (7.8%)	
	Other	1257 (18.3%)	401 (22.9%)	671 (16.5%)	185 (17.7%)	

Acute care utilization in the last year of life (truncating the last 30 days of life) varied from the last month of life. Patients who received a Primary Care, Palliative Care, and Nephrology care triad had the highest percentages of any hospitalization (84.3%) or any emergency department visits (91.6%) in the 11 months before the last month of life (Table 3), compared to patients who received other patterns of care. However, patients who received the care triad also spent the most days in hospital or emergency department in the last year of life (median [IQR]: 26 [8-56]), compared to the primary care and nephrology dyad (median [IQR]: 16 [4-44]) or the non-primary care pattern (median [IQR]: 15 [2-49]). All differences across the last year of life were also statistically significant (<.05).

## Discussion

### Main Findings

In this study of patients who died with KF on maintenance dialysis in Ontario, Canada, patients received most of their outpatient care from nephrologists in the last year of life. This finding is unsurprising as patients with kidney failure who receive in-center hemodialysis receive dialysis 3 times a week and see a nephrologist once a week.^29^ Therefore, their outpatient care is anchored to their dialysis treatment, and therefore, they have high continuity with their nephrology team.^4^ Among patients who experienced primary care encounters, primary care physicians were also consistently involved in the last year of life, albeit on a smaller scale compared to nephrologists. This phenomenon may be specific to patients with KF on maintenance dialysis as primary care plays a much more prominent role compared to specialists in end-of-life outpatient care among the general decedent population.^9^ Patients with KF on maintenance dialysis have traditionally looked to their nephrology team for advance care planning, symptom management, and social and emotional support.^30^ This relationship is appropriate; however, primary care physicians may also be well-suited to provide these services due to their longitudinal relationships with their patients.^31^ Given the potential benefits of primary care involvement in providing end-of-life care, it is notable that almost 15.5% of patients did not have any encounters with primary care during the last year of life; care was managed primarily by their dialysis team in these cases. Patients who wish to discontinue dialysis may experience a disconnect in care if primary care is not involved as they navigate non-dialysis-related healthcare services. Overall, most patients had continued contact with their primary care physician during their last year of life, in addition to other specialties.

Palliative care physician involvement increased as patients approached death, with large increases occurring in the last 3 months of life. Specialist palliative care is not being initiated early in the last year of life and is concentrated near death for patients dying with KF on maintenance dialysis. This finding aligns with other studies that identified that patients on dialysis access palliative care late in the end-of-life period.^1,2,32,33^ Patients with primary care involvement experienced greater palliative care involvement from both provincial home care services and palliative care physicians, suggesting that patients without primary care involvement may experience barriers to palliative care access or that nephrologists are providing end-of-life care for their patients. Furthermore, almost 1 in 5 monthly encounters were attributed to other specialists, beyond primary care, nephrology, or palliative care. Therefore, patients dying with KF on maintenance dialysis are experiencing multiple comorbidities that require medical attention from other specialists, resulting in more outpatient encounters during the last year of life.

End-of-life healthcare utilization did not vary extensively for patients dying with KF on maintenance dialysis across patterns of care. Almost 80% of patients in each pattern of care experienced at least one hospitalization or emergency department visit in the last 30 days of life—which is higher than previously reported research.^1^ Patients dying with KF on maintenance dialysis may require more inpatient care provision at the end of life than other populations due to complications associated with reduced intervention effectiveness or other comorbidities (i.e., myocardial infarctions and infections). Patients who had palliative care physician involvement experienced lower rates of death in acute care settings, despite being more medically complex, as demonstrated by higher percentages of home care services enrolment, cancer prevalence, and spending more days in hospital or emergency departments in the last 30 days of life and having the greatest acute care utilization in the 11 months before the last 30 days of life. These findings complement existing literature that identified that patients on dialysis who received home palliative care services were less likely to die in acute or sub-acute care settings compared to those who did not receive home palliative care services.^1^ Therefore, involving palliative care physicians may facilitate patients being discharged from inpatient settings to pass away in their homes, which is often the preferred location of death for patients.^34-37^ However, the timing and rationale of palliative care involvement remain uncertain, and future research ought to explore the characteristics associated with earlier palliative care referral.

### Strengths and Limitations

This project has strengths, such as leveraging existing routinely collected health administrative data from Ontario, Canada. This data includes deidentified patient-level health information from approximately 13 million people with access to near-universal healthcare. As such, our findings may be relevant to other jurisdictions with public healthcare systems that also provide strong primary care services to their patients. This study also has limitations. First, we relied on different definitions for outpatient encounters with nephrology, as nephrologists do not typically bill for individual encounters for patients with KF on maintenance dialysis. As such, we may be overestimating the number of outpatient encounters that patients experience with their nephrologists by relying on individual encounter billing codes and weekly dialysis management billing codes. Some patients may receive primary care from nurse practitioners who do not have capturable OHIP physician billing codes. Therefore, patients in the non-primary care pattern may still benefit from having a primary care provider involved. Similarly, patients in the primary care and nephrology care dyad may have received palliative care services from their primary care physician or nephrologist. We also excluded patients who resided in long-term care homes during the last year of life as they may experience different outpatient care patterns compared to patients residing in the community. However, approximately 5% of chronic dialysis patients reside in long-term care homes in Ontario, and more long-term care homes have received funding to deliver peritoneal dialysis between 2012 and 2019,^38^ and therefore, the number of patients receiving dialysis in long-term care is expected to increase. Next, our study only includes data before the COVID-19 pandemic, and therefore, may not reflect contemporary outpatient patterns. Lastly, this was a descriptive study, and therefore, cannot infer causal relationships between patterns of outpatient physician care and end-of-life healthcare utilization as there is temporal ambiguity. As a result, a separate study will be conducted to measure the associations between patterns of physician care and end-of-life healthcare outcomes, adjusting for patients’ demographic and clinical characteristics.

## Conclusions

The end-of-life experience for patients dying with KF on maintenance dialysis in Ontario, Canada, heavily relies on nephrologists. Dialysis provision ensures patients experience high continuity of care with their nephrology care team, while primary care also remains a consistent, but lesser, presence. Acute care utilization during the last month of life is consistent across all patients with KF on maintenance dialysis regardless of the pattern of care, although patients are less likely to die in acute care settings when palliative care is involved. Future research ought to identify the associations between the identified patterns of outpatient care and end-of-life healthcare outcomes to identify the most optimal physician pattern of care for patients with KF on maintenance dialysis. As such, this endeavor may provide further justification for improved collaboration between nephrology, primary care and palliative care for patients at the end of life.

## Supplemental Material

sj-docx-1-cjk-10.1177_20543581241280698 – Supplemental material for End-of-Life Care Among Patients With Kidney Failure on Maintenance Dialysis: A Retrospective Population-Based Study Supplemental material, sj-docx-1-cjk-10.1177_20543581241280698 for End-of-Life Care Among Patients With Kidney Failure on Maintenance Dialysis: A Retrospective Population-Based Study by Shuaib Hafid, Sarina R. Isenberg, Aleisha Fernandes, Erin Gallagher, Colleen Webber, Meera Joseph, Manish M Sood, Adrianna Bruni, Janet L. Davis, Grace Warmels, James Downar, Anastasia Gayowsky, Aaron Jones, Doug Manuel, Peter Tanuseputro and Michelle Howard in Canadian Journal of Kidney Health and Disease

sj-docx-2-cjk-10.1177_20543581241280698 – Supplemental material for End-of-Life Care Among Patients With Kidney Failure on Maintenance Dialysis: A Retrospective Population-Based Study Supplemental material, sj-docx-2-cjk-10.1177_20543581241280698 for End-of-Life Care Among Patients With Kidney Failure on Maintenance Dialysis: A Retrospective Population-Based Study by Shuaib Hafid, Sarina R. Isenberg, Aleisha Fernandes, Erin Gallagher, Colleen Webber, Meera Joseph, Manish M Sood, Adrianna Bruni, Janet L. Davis, Grace Warmels, James Downar, Anastasia Gayowsky, Aaron Jones, Doug Manuel, Peter Tanuseputro and Michelle Howard in Canadian Journal of Kidney Health and Disease

sj-docx-3-cjk-10.1177_20543581241280698 – Supplemental material for End-of-Life Care Among Patients With Kidney Failure on Maintenance Dialysis: A Retrospective Population-Based Study Supplemental material, sj-docx-3-cjk-10.1177_20543581241280698 for End-of-Life Care Among Patients With Kidney Failure on Maintenance Dialysis: A Retrospective Population-Based Study by Shuaib Hafid, Sarina R. Isenberg, Aleisha Fernandes, Erin Gallagher, Colleen Webber, Meera Joseph, Manish M Sood, Adrianna Bruni, Janet L. Davis, Grace Warmels, James Downar, Anastasia Gayowsky, Aaron Jones, Doug Manuel, Peter Tanuseputro and Michelle Howard in Canadian Journal of Kidney Health and Disease

sj-docx-4-cjk-10.1177_20543581241280698 – Supplemental material for End-of-Life Care Among Patients With Kidney Failure on Maintenance Dialysis: A Retrospective Population-Based Study Supplemental material, sj-docx-4-cjk-10.1177_20543581241280698 for End-of-Life Care Among Patients With Kidney Failure on Maintenance Dialysis: A Retrospective Population-Based Study by Shuaib Hafid, Sarina R. Isenberg, Aleisha Fernandes, Erin Gallagher, Colleen Webber, Meera Joseph, Manish M Sood, Adrianna Bruni, Janet L. Davis, Grace Warmels, James Downar, Anastasia Gayowsky, Aaron Jones, Doug Manuel, Peter Tanuseputro and Michelle Howard in Canadian Journal of Kidney Health and Disease
