# Supplementary material for: End-of-Life Care Among Patients With Kidney Failure on Maintenance Dialysis: A Retrospective Population-Based Study
Source: Can J Kidney Health Dis. 2024 Sep 21;11:20543581241280698. doi: 10.1177/20543581241280698 (PMC11418337; doi:10.1177/20543581241280698)
Supplement: sj-docx-1-cjk-10.1177_20543581241280698 – Supplemental material for End-of-Life Care Among Patients With Kidney Failure on Maintenance Dialysis: A Retrospective Population-Based Study [file sj-docx-1-cjk-10.1177_20543581241280698.docx]

**Supplementary File 1:** Description of health administrative data files held at ICES used in the study.

| **DATABASE** | **DATABASE CONTENT** |
| --- | --- |
| ICES-derived cohorts | Validated cohorts of individuals with specific diseases and conditions. These include the Ontario Asthma Dataset (ASTHMA); Congestive Heart Failure (CHF) database; Chronic Obstructive Pulmonary Disease (COPD) database; Ontario Dementia Dataset (DEMENTIA); Ontario Hypertension Dataset (HYPER); Ontario Crohn’s and Colitis Cohort Dataset (OCCC); Ontario Diabetes Dataset (ODD); Ontario Myocardial Infarction Dataset (OMID); and the Ontario Rheumatoid Arthritis Dataset (ORAD). |
| Ontario Health Insurance Plan database (OHIP) | This dataset includes all claims by Ontario physicians for inpatient and ambulatory visits, consultations, and procedures. The data also include claims from optometrists for publicly funded reimbursement and from laboratories for all diagnostic tests performed. |
| Ontario Registered Persons Database (RPDB) | Demographic, place of residence and vital status information for all persons eligible to receive insured health services in the province, including date of birth, sex, and home address. |
| Client Agency Program Enrolment (CAPE) Dataset | This dataset details a list of patients registered to a primary care organization and identifies an association with a specific primary care physician and what type of primary care organization. |
| Canadian Organ Replacement Register (CORR) | This database collects data from dialysis programs, and organ transplant lists and procedures. |
| Discharge Abstract Database (DAD) | This database contains administrative, clinical, and demographic information on all hospital discharges. |
| National Ambulatory Care Reporting System (NACRS) | This database contains administrative, clinical, and demographic information for all hospital and community-based ambulatory care provision, including emergency departments. |
| Home Care Database (HCD) | This database contains administrative, clinical, and demographic information for patients accessing provincial homecare services. |
