# Supplementary material for: End-of-Life Care Among Patients With Kidney Failure on Maintenance Dialysis: A Retrospective Population-Based Study
Source: Can J Kidney Health Dis. 2024 Sep 21;11:20543581241280698. doi: 10.1177/20543581241280698 (PMC11418337; doi:10.1177/20543581241280698)
Supplement: sj-docx-2-cjk-10.1177_20543581241280698 – Supplemental material for End-of-Life Care Among Patients With Kidney Failure on Maintenance Dialysis: A Retrospective Population-Based Study [file sj-docx-2-cjk-10.1177_20543581241280698.docx]

**Supplementary File 2:** Defining outpatient nephrology encounters.

| **Method** | **Source** | **Description** |
| --- | --- | --- |
| 1 | Ontario Health Insurance Billing Codes | Billing codes where the specialty code is Nephrology, and the location variable is Home, Office, or Phone. |
| 2 | Ontario Health Insurance Billing Codes | Dialysis weekly billing codes of G860, G861, G862, G863, G864, G865, or G866. |
