# Supplementary material for: End-of-Life Care Among Patients With Kidney Failure on Maintenance Dialysis: A Retrospective Population-Based Study
Source: Can J Kidney Health Dis. 2024 Sep 21;11:20543581241280698. doi: 10.1177/20543581241280698 (PMC11418337; doi:10.1177/20543581241280698)
Supplement: sj-docx-3-cjk-10.1177_20543581241280698 – Supplemental material for End-of-Life Care Among Patients With Kidney Failure on Maintenance Dialysis: A Retrospective Population-Based Study [file sj-docx-3-cjk-10.1177_20543581241280698.docx]

**Supplementary File 3**: Individual-level monthly percentages of outpatient encounters to different specialties during the last year of life among patients dying with KF on maintenance dialysis between Jan. 1, 2017, and Dec. 31, 2019, in Ontario, Canada (Tabular Format).

| **Pattern** | **Specialty** | **Mean Percentage of Outpatient Encounters** | | | | | | | | | | | |
| --- | --- | --- | --- | --- | --- | --- | --- | --- | --- | --- | --- | --- | --- |
|  |  | **12 - 11 Months** | **11 - 10 Months** | **10 - 9 Months** | **9 - 8 Months** | **8 - 7 Months** | **7 - 6 Months** | **6 - 5 Months** | **5 - 4 Months** | **4 - 3 Months** | **3 - 2 Months** | **2 - 1 Months** | **Last Month** |
| Total Cohort | Primary Care | 14% | 14% | 14% | 13% | 13% | 13% | 12% | 11% | 11% | 10% | 10% | 11% |
|  | Palliative care | 1% | 1% | 1% | 1% | 1% | 1% | 2% | 2% | 2% | 2% | 3% | 10% |
|  | Nephrology | 64% | 65% | 65% | 66% | 66% | 67% | 68% | 69% | 68% | 69% | 69% | 61% |
|  | Internal Medicine | 3% | 3% | 3% | 3% | 3% | 3% | 3% | 3% | 3% | 3% | 3% | 4% |
|  | Oncology | 1% | 1% | 1% | 1% | 1% | 1% | 1% | 1% | 1% | 1% | 1% | 1% |
|  | Other | 16% | 16% | 16% | 16% | 16% | 15% | 15% | 15% | 15% | 14% | 14% | 13% |
| Pattern: Primary Care, Palliative Care, Nephrology Triad | Primary Care | 17% | 18% | 17% | 17% | 16% | 16% | 15% | 14% | 13% | 12% | 11% | 11% |
|  | Palliative care | 3% | 3% | 4% | 4% | 4% | 4% | 5% | 5% | 6% | 8% | 10% | 33% |
|  | Nephrology | 56% | 56% | 56% | 56% | 57% | 58% | 59% | 60% | 60% | 60% | 60% | 43% |
|  | Internal Medicine | 3% | 3% | 3% | 2% | 3% | 3% | 3% | 3% | 3% | 3% | 3% | 3% |
|  | Oncology | 2% | 1% | 2% | 2% | 2% | 2% | 2% | 2% | 2% | 3% | 2% | 1% |
|  | Other | 18% | 18% | 18% | 18% | 18% | 17% | 16% | 16% | 15% | 15% | 14% | 9% |
| Pattern: Primary Care, Nephrology Dyad | Primary Care | 16% | 16% | 17% | 15% | 15% | 15% | 14% | 13% | 13% | 12% | 11% | 14% |
|  | Palliative care | 0% | 0% | 0% | 0% | 0% | 0% | 0% | 0% | 0% | 0% | 0% | 0% |
|  | Nephrology | 63% | 64% | 64% | 65% | 66% | 67% | 67% | 68% | 68% | 70% | 70% | 66% |
|  | Internal Medicine | 3% | 3% | 3% | 3% | 3% | 3% | 3% | 3% | 3% | 3% | 3% | 5% |
|  | Oncology | 0% | 0% | 0% | 0% | 0% | 0% | 0% | 0% | 0% | 1% | 0% | 0% |
|  | Other | 17% | 16% | 16% | 16% | 16% | 15% | 15% | 16% | 15% | 15% | 15% | 15% |
| Pattern: Non-Primary Care | Primary Care | 0% | 0% | 0% | 0% | 0% | 0% | 0% | 0% | 0% | 0% | 0% | 0% |
|  | Palliative care | 1% | 1% | 1% | 2% | 1% | 2% | 2% | 2% | 2% | 2% | 4% | 9% |
|  | Nephrology | 83% | 84% | 84% | 84% | 84% | 85% | 84% | 84% | 84% | 83% | 83% | 74% |
|  | Internal Medicine | 3% | 3% | 3% | 2% | 3% | 3% | 2% | 2% | 2% | 3% | 3% | 4% |
|  | Oncology | 1% | 1% | 1% | 0% | 1% | 0% | 1% | 1% | 1% | 1% | 0% | 0% |
|  | Other | 12% | 11% | 11% | 12% | 11% | 10% | 11% | 11% | 11% | 10% | 10% | 12% |
